# MiR-320a acts as a prognostic factor and Inhibits metastasis of salivary adenoid cystic carcinoma by targeting *ITGB3*

**DOI:** 10.1186/s12943-015-0344-y

**Published:** 2015-04-29

**Authors:** Lijuan Sun, Bodu Liu, Zhaoyu Lin, Yandan Yao, Yanyang Chen, Yang Li, Jianing Chen, Dongsheng Yu, Zhangui Tang, Bosheng Wang, Shuguang Zeng, Song Fan, Youyuan Wang, Yilin Li, Erwei Song, Jinsong Li

**Affiliations:** Guangdong Provincial Key Laboratory of Malignant Tumor Epigenetics and Gene Regulation, Sun Yat-Sen Memorial Hospital, Sun Yat-Sen University, Guangzhou, 510120 China; Department of Oral & Maxillofacial Surgery, Sun Yat-sen Memorial Hospital, Sun Yat-sen University, Guangzhou, 510120 China; Breast Tumor Center, Sun Yat-sen Memorial Hospital, Sun Yat-sen University, Guangzhou, 510120 China; Department of Pathology, the First Affiliated Hospital, Sun Yat-sen University, Guangzhou, 510080 China; Guanghua School of Stomatology, Sun Yat-sen University, Guangzhou, 510055 China; Xiangya School of Stomatology, Central South University, Changsha, 410078 China; Guangdong Provincial Stomatological Hospital, Guangzhou, 510280 China; Xaverian Brothers High School, Westwood, MA 02090 USA; Sun Yatsen Memorial Hospital, Sun Yat-sen University, Guangzhou, 510120 China

**Keywords:** Metastasis, miR-320a, *ITGB3*, Adenoid cystic carcinoma, Prognosis

## Abstract

**Background:**

Salivary Adenoid cystic carcinoma (SACC) patients with local invasion and lung metastasis are often resistant to conventional therapy such as operation, chemotherapy and radiotherapy. To explore the underling mechanisms, we studied the roles of miRNA in regulating invasiveness of SACC cells.

**Methods:**

MicroRNA profiling was done in SACC cells with microarray. MiRNA mimics or antisense oligonucleotide was transfected and invasiveness of SACC cells was evaluated by adhesion assay and transwell assay. The target gene of miRNA was identified by luciferase reporter assay and “rescue” experiment. Tumor metastasis was evaluated by BALB/c-nu mice xenografts. MiRNA and its target gene expression were identified by in-situ hybridization and immunohistochemistry respectively, in 302 patients from affiliated hospitals of Sun Yat-sen University and in 148 patients from affiliated hospitals of Central South University, and correlated to the clinicopathological status of the patients.

**Results:**

MiR-320a was down-regulated in high lung metastatic ACCM and SACC-LM cells compared with the corresponding low metastatic ACC2 and SACC-83 cells, and inhibited adhesion, invasion and migration of SACC cells by targeting integrin beta 3 (*ITGB3*). *In vivo*, enforced miR-320a expression suppressed metastasis of SACC xenografts. In the two independent sets, miR-320a was downregulated in primary SACCs with metastasis compared to those without metastasis, and low expression of this miRNA predicts poor patient survival and rapid metastasis. Multivariate analysis showed that miR-320a expression was an independent indicator of lung metastasis.

**Conclusions:**

MiR-320a inhibits metastasis in SACCs by targeting *ITGB3* and may serve as a therapeutic target and prognostic marker in salivary cancers.

**Electronic supplementary material:**

The online version of this article (doi:10.1186/s12943-015-0344-y) contains supplementary material, which is available to authorized users.

## Background

Salivary adenoid cystic carcinoma (SACC) mainly occurs in the salivary duct, accounting for 24% of malignant salivary gland tumors. SACC exhibits certain unique characteristics, such as neurotropic, infiltrative growth and distant metastasis [1-3]. For SACC patients, distant metastasis is a crucial prognostic factor [4,5]. Therefore, determining the mechanisms that govern the perineural invasion and metastasis of SACCs is essential for the development of novel therapeutic strategies to improve patient survival.

MicroRNAs (miRNAs) are a class of small non-coding RNAs (~22 nucleotides in length) that are endogenously expressed in mammalian cells. miRNAs regulate gene expression by repressing mRNA translation or cleaving target mRNAs. miRNAs may function as oncogenes or tumor suppressors by modulating multiple cellular pathways, including cell proliferation [6], differentiation [7,8], apoptosis [9,10], and invasion [11-13]. There are significant differences in the miRNA expression profiles of a variety of human cancers, including colon, breast, lung, and stomach cancer, as well as lymphoma and leukemia, compared with the corresponding normal tissues [6,7,14]. Recently, a study found that 19 miRNAs were upregulated and 36 miRNAs were downregulated in primary SACC specimens, compared with matched normal samples [15]. However, the mechanisms by which miRNAs regulate the biological characteristics of SACC remains unclear.

Integrins are a family of transmembrane receptors that mediate the attachment between a cell and its surroundings, such as other cells or the extracellular matrix (ECM), and play important roles in signal transduction [16-19]. Integrins regulate cell proliferation, survival and migration mainly through FAK (focal adhesion kinase) and Src kinase family members [20,21]. Functional integrins are heterodimers containing two distinct subunits, α and β. There are 18 α subunits and 8 β subunits found in mammals. Heterodimers formed between different subunits have different structures and functions [17,18]. It has been reported in breast, colorectal cancer and hepatocellular carcinoma that dysfunctions in microRNA-regulated integrin signaling were involved in tumor metastasis and apoptosis [10,22,23].

In the present study, we screened for differentially expressed miRNAs in high lung metastatic SACC cells compared with their corresponding low metastatic cells and identified miR-320a as a metastatic repressor. We then explored the mechanism by which miR-320a regulates the invasiveness of SACC cells and the metastasis of SACC xenografts and identified its functional target as integrin beta 3 (*ITGB3*). Finally, we found significant correlations between miR-320a expression and the clinicopathological status and prognosis of SACC patients in two independent sets. These findings have advanced our knowledge of the molecular mechanisms of SACC metastasis and provided potential markers and therapeutic targets for the diagnosis and treatment of SACC.

## Results

### Reduction of miR-320a promotes invasiveness of salivary adenoid cystic carcinoma cells

**Figure 1:**
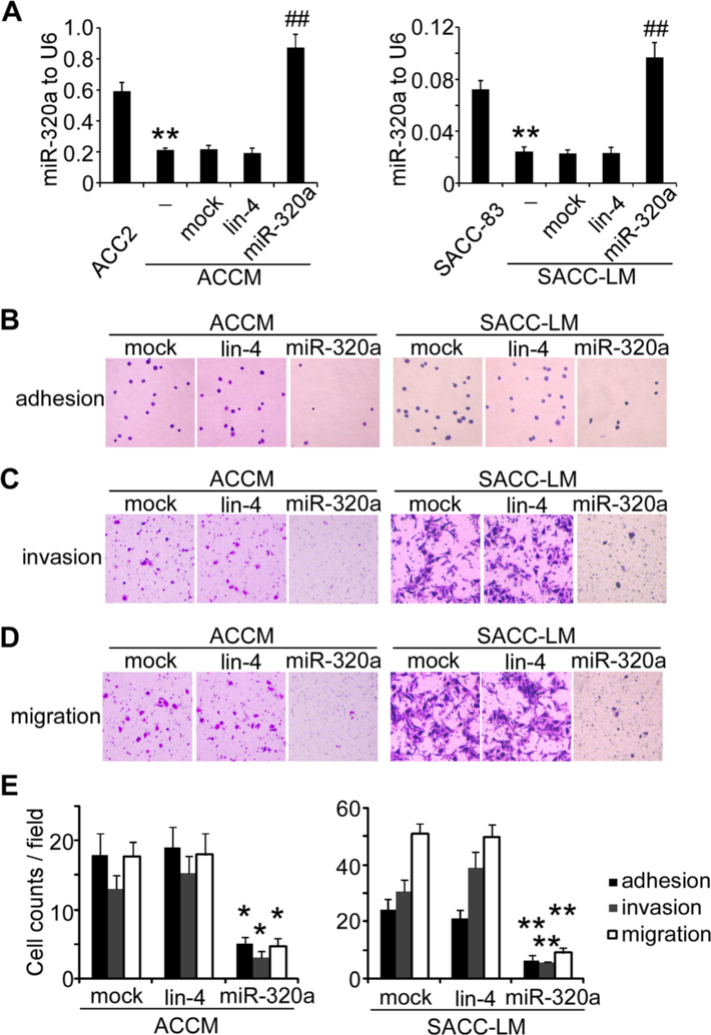
Ectopic expression of miR-320a inhibits the invasiveness of salivary adenoid cystic carcinoma cells. **(A)** miR-320a expression in SACC cells was determined by qRT-PCR analysis. **P < 0.01 vs. parental SACC cells. ^##^ *P* < 0.01 vs. mock transfection. U6 was used as an internal control. Adhesion assays **(B)** and transwell assays **(C, D)** showed that adhesion, invasion and migration of ACCM and SACC-LM cells were inhibited by miR-320a mimics (100×). **(E)** Quantification of adhesive, invasive and migratory cells assessed by adhesion assays and transwell assays. *P < 0.05; **P < 0.01 vs. mock.

To identify miRNAs involved in SACC metastasis, we screened for miRNAs that are differentially expressed in high lung metastatic ACCM cells and the parental low metastatic ACC2 cells via microarray analysis (Additional file 1). qRT-PCR further validated miR-320a as the most significantly downregulated miRNA in ACCM cells (Additional file 2). Transfection of the high lung metastatic cells with miR-320a mimics but not the non-relevant lin4 mimics specifically increased miR-320a levels in ACCM and SACC-LM cell (*P* < 0.01, Figure 1A).

We next investigated whether ectopic expression of miR-320a inhibited the invasiveness in SACC cells. Transfection of miR-320a mimics remarkably inhibited the adhesion of ACCM cells (Figure 1B and E, *P* < 0.05). Transwell assays also demonstrated that miR-320a inhibited ACCM cell invasion and migration by 72% (Figure 1C and E, *P* < 0.05) and 60% (Figure 1D and E, *P* < 0.05), respectively. Transfection with miR-320a also inhibited SACC-LM cell adhesion, invasion and migration by 75% (Figure 1B and E, *P* = 0.001), 80% (Figure 1C and E, *P* = 0.008) and 82% (Figure 1D and E, *P* = 0.007), respectively.

In contrast, silencing miR-320a expression in the parental ACC2 and SACC-83 cells using antisense oligonucleotides (ASO) enhanced the invasiveness of these cells (Additional file 3). The adhesion, invasion and migration of ACC2 cells increased by 2-, 3-, and 2.9-fold, respectively, and that of SACC-83 cells increased by 2.2-, 4.7- and 7.2-fold, respectively. Therefore, reduced miR-320a levels contribute to enhanced invasiveness in SACC cells.

### miR-320a inhibits the invasiveness of SACC cells by targeting *ITGB3*

**Figure 2:**
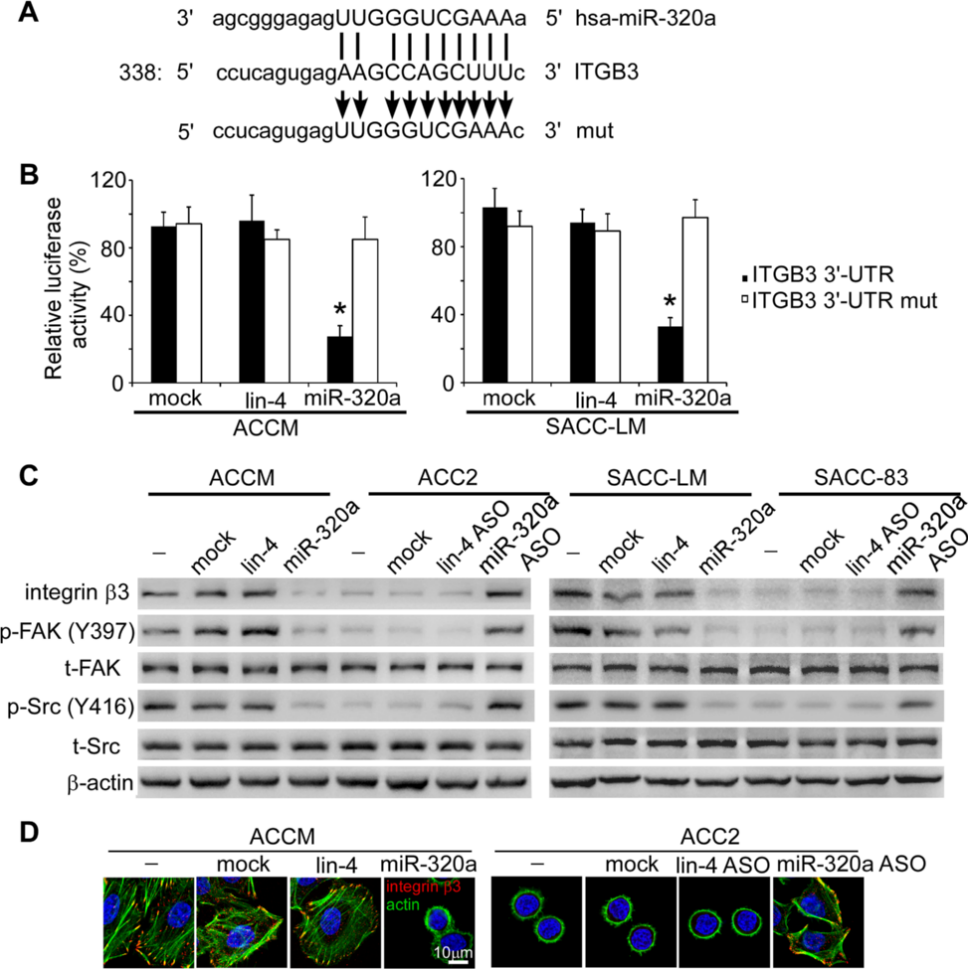
*ITGB3* is an miR-320a target gene. **(A)** Target sequence of miR-320a in *ITGB3* 3′-UTR predicted by TargetScan, as well as the mutated sequence. **(B)** Luciferase reporter assays were performed in ACCM and SACC-LM cells co-transfected with miR-320a-mimics and reporter vectors carrying a *ITGB3* 3′-UTR with a wild-type (*ITGB3* 3′-UTR) or mutated miR-320a (*ITGB3* 3′-UTR mut) response element. *P < 0.05 vs. mock transfection. **(C)** Integrin β3 expression, as well as FAK and Src phosphorylation, was assessed using western blot analysis. β-actin was used as an internal control. **(D)** Colocalization of integrin β3 and the cytoskeletal protein actin was illustrated by immunofluorescence staining. Nuclei: blue, scale bar: 10 μm.

miRNAs act by binding to the 3′-untranslated region (3′-UTR) of target genes through partial sequence homology. We predicted the target genes of miR-320a using TargetScan and found an miR-320a recognition site in the 3′-UTR of *ITGB3* (Figure 2A). To confirm whether *ITGB3* is a target of miR-320a, we performed luciferase reporter assays and evaluated the relative luciferase activities in SACC cells transfected with a reporter plasmid carrying an miR-320a target sequence (*ITGB3* 3′-UTR) versus those transfected with a control plasmid. It was shown that co-transfection with miR-320a mimics significantly reduced the luciferase activity in ACCM and SACC-LM cells (Figure 2B). However, when the miRNA target sequence was mutated in the reporter plasmids (*ITGB3* 3′-UTR mut), transfection with miR-320a mimics failed to influence the relative luciferase activity, suggesting that miR-320a suppresses *ITGB3* expression in SACC cells.

To study whether *ITGB3* serves as a functional target of miR-320a, we examined integrin β3 expression in these cells. Western blot analysis showed that integrin β3 expression in ACCM and SACC-LM cells was much higher than in the parental ACC2 and SACC-83 cells (Figure 2C). Transfection with miR-320a mimics reduced integrin β3 expression in ACCM and SACC-LM cells, while transfection with the miR-320a ASO enhanced integrin β3 expression in the parental ACC2 and SACC-83 cells. Furthermore, FAK and Src phosphorylation was suppressed by the miR-320a mimics, and enhanced by the miR-320a ASO, suggesting that miR-320a regulates the activity of the integrin signaling pathway. Furthermore, immunofluorescence staining demonstrated that the cytoskeletal protein actin accumulated in clusters and co-localized with clustered integrin β3 on the ACCM cell surface (Figure 2D). These phenotypes were regulated by miR-320a because transfecting ACCM cells with miR-320a mimics resulted in the downregulation of integrin β3 and a ring-like distribution of actin. In contrast, in ACC2 cells, miR-320a ASO promoted the aggregation of actin and its co-localization with clustered integrin β3 on the cell surface.

**Figure 3:**
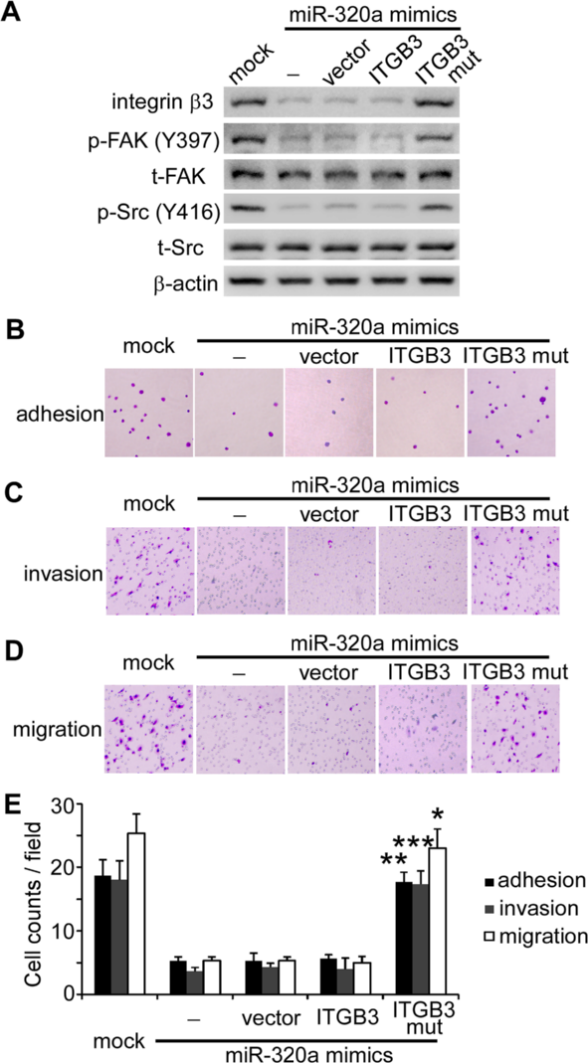
miR-320a inhibits the invasiveness of SACC cells by targeting *ITGB3*. **(A)** Integrin β3 expression and FAK and Src phosphorylation were determined by western blot analysis in ACCM cells transfected with miR-320a mimics alone or in combination with either pcDNA 3.1+ (Vector) or pcDNA 3.1+ containing a wild-type (*ITGB3*) or mutant *ITGB3* expression cassette of the miR-320a (*ITGB3* mut) response element. β-actin was used as an internal control. Adhesion, invasion and migration of ACCM cells were assessed using adhesion assays **(B)** and transwell assays **(C, D)** (100x). **(E)** Quantification of adhesive, invasive and migratory ACCM cells. *P < 0.05; **P < 0.01; ***P < 0.001 vs. vector.

To identify whether miR-320a inhibits the invasiveness of SACC cells by targeting *ITGB3*, we performed a “rescue” experiment by co-transfecting ACCM cells with miR-320a mimics and a pcDNA3.1 vector that expresses *ITGB3* with mutated miR-320a seed sequence (*ITGB3* mut) in the 3′-UTR. Western blot analysis demonstrated that co-transfection of ACCM cells with miR-320a mimics and *ITGB3* mut restored integrin β3 expression, as well as FAK and Src phosphorylation, whereas co-transfecting wild-type pcDNA3.1-*ITGB3* could not restore integrin β3 expression after silencing by miR-320a mimics (Figure 3A). Moreover, “rescuing” integrin β3 expression in the presence of miRNA mimics restored adhesion, invasion and migration of ACCM cells (Figure 3, B-E). Collectively, these data suggest that miR-320a regulates the invasiveness of SACC cells by targeting *ITGB3*.

### Enforced miR-320a expression inhibits ACCM xenograft metastasis

Given that miR-320a expression inhibits the invasiveness of SACC cells *in vitro*, we further assessed its effect on metastasis *in vivo*. miR-320a was stably expressed in ACCM cells by transduction with an miRNA-expressing vector (EmGFP-320a, Additional file 4A). As shown in Additional file 4B, enforced miR-320a expression did not alter tumor growth in BALB/c-nu mice inoculated with ACCM cells.

**Figure 4:**
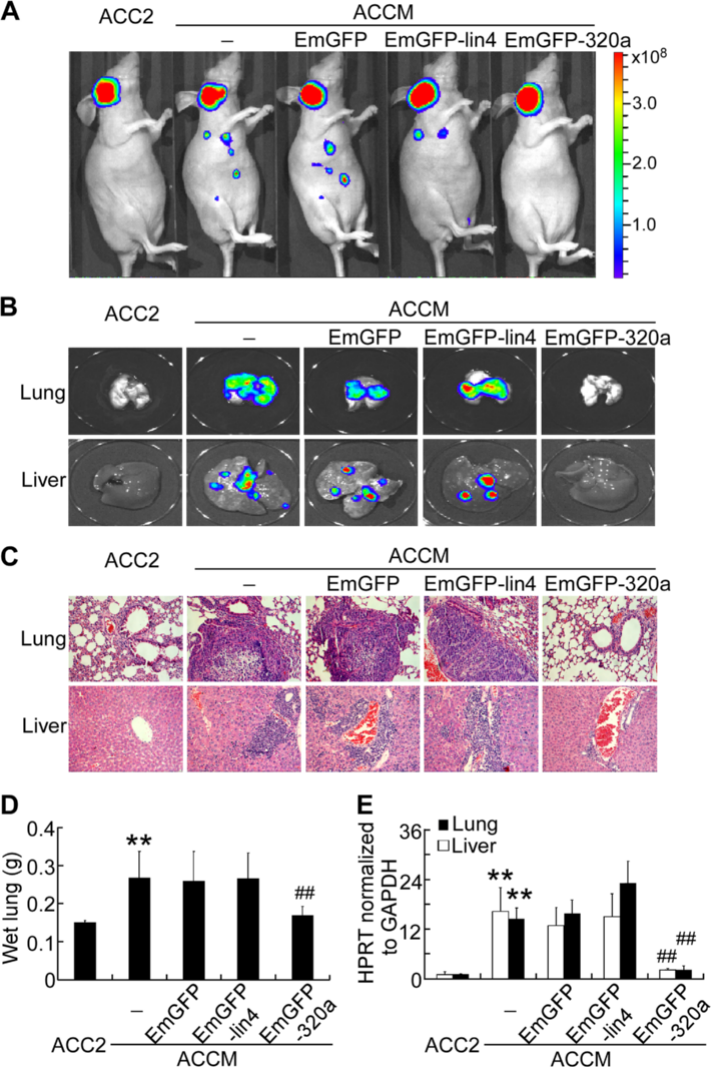
Ectopic expression of miR-320a inhibits ACCM xenograft metastasis in BALB/c-nu mice. **(A)** Whole-body luminal imaging was performed in mice inoculated with ACCM stably expressing the luciferase gene. Luminal images **(B)** and HE staining **(C)** of paraffin sections (200×) of the lungs (upper) and livers (lower) of the tumor-bearing mice. **(D)** Mean ± SD wet lung weight of tumor-bearing mice (n = 8/group). **(E)** Expression of human HPRT mRNA relative to mouse 18S rRNA in the lungs and livers of the tumor-bearing mice was determined by qRT-PCR analysis. **P < 0.01 vs. ACC2 cells. ^##^ *P* < 0.01 vs. EmGFP transfection.

However, luminescence imaging of the whole body (Figure 4A) and harvested lungs and livers (Figure 4B) demonstrated that ACCM cells metastasized to the lungs and livers of tumor-bearing mice, and miR-320a expression suppressed this metastasis. HE staining also revealed that miR-320a expression inhibited metastasis in the lungs and livers of mice bearing ACCM xenografts compared with non-relevant miRNA (Figure 4C). Furthermore, the average lung weight of ACCM tumor-bearing mice was reduced by miR-320a expression (Figure 4D, n = 8, *P* < 0.01). The number of metastasized tumor cells, as quantified by qRT-PCR for human HPRT in tumor-bearing mice, was reduced by 80% and 70% in the lungs and livers, respectively, of animals inoculated with miR-320a-expressing ACCM cells (Figure 4E, *P* < 0.01). HE staining of the xenografts also indicated that the focal metastasis of ACCM tumors was markedly reduced by miR-320a (Additional file 4C). Therefore, miR-320a overexpression significantly suppressed the metastasis of ACCM xenografts.

Moreover, immunohistochemical staining revealed that miR-320a overexpression reduced integrin β3 expression by more than 60% (Additional file 4C and D) but did not influence the percentage of proliferative PCNA^+^ tumor cells (Additional file 4C and E). These findings indicate that miR-320a inhibits SACC metastasis *in vivo*, most likely by silencing *ITGB3*.

### Low miR-320a expression indicates poor patient survival and high SACC metastasis

**Figure 5:**
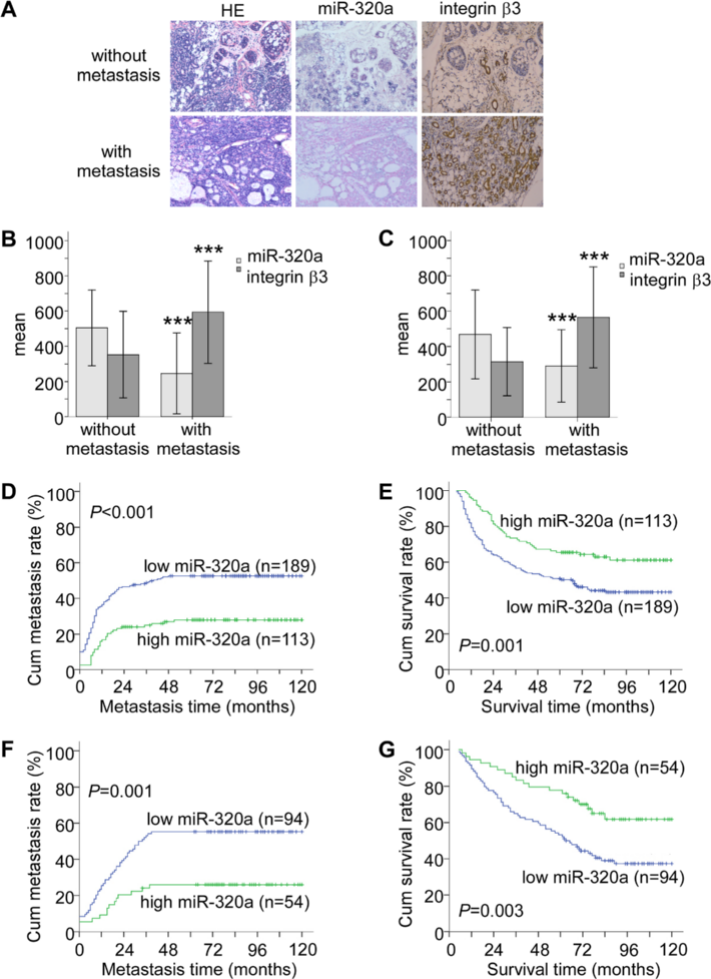
miR-320a downregulation correlates with metastasis and poor patient survival in SACCs. **(A)** *In situ* hybridization for miR-320a and immunohistochemical staining for integrin β3 in primary SACCs with metastasis vs. those without metastasis (200×). Quantification of miR-320a and integrin β3 expression in primary SACC samples from the affiliated hospitals of Sun Yat-sen University **(B)** or from the affiliated hospitals of Central South University **(C)**. ***P < 0.001. Kaplan-Meier curves for SACC patients from the affiliated hospitals of Sun Yat-sen University comparing miR-320a expression and metastasis **(D)** or miR-320a expression and survival **(E)** was analyzed by the log rank test. Kaplan-Meier curves for SACC patients from the affiliated hospitals of Central South University comparing miR-320a expression and metastasis **(F)** or miR-320a expression and survival **(G)**. The median value was used to distinguish high expression and low expression.

We further evaluated the clinical significance of miR-320a expression in patient prognosis and SACC metastasis. *In situ* hybridization and immunohistochemical staining (Figure 5A) demonstrated that miR-320a expression was lower and integrin β3 expression was higher in primary SACCs with metastasis compared with those without metastasis. The difference in expression between SACCs with metastasis and SACCs without metastasis from the affiliated hospitals of Sun Yat-sen University was statistically significant (Figure 5B, *P* < 0.001). Spearman order correlation analysis showed that integrin β3 expression in SACCs was inversely correlated with the miR-320a level (Additional file 5A, *r*_*s*_ = −0.839, *P* < 0.001). Furthermore, SACCs with metastasis collected at affiliated hospitals of Central South University had remarkably low miR-320a expression and high integrin β3 expression (Figure 5C, *P* < 0.001), and the integrin β3 expression in the SACC paitents was negatively correlated with miR-320a levels (Additional file 5B, *r*_*s*_ = −0.857, *P* < 0.001).

Next, we analyzed the association between miR-320a expression and the clinicopathologic status of SACC patients (Table 1). No significant correlation was observed between miR-320a expression and age, sex, tumor size or TNM stage. However, the miR-320a levels were closely associated with distant metastasis in patients from the two independent sets (*P* < 0.001, or *P* = 0.001, respectively). Tumors with distant metastasis expressed low levels of miR-320a. Conversely, integrin β3 expression was positively correlated with distant metastasis in SACC patients.

**Table 1:** **Correlation among clinicopathologic status and the expression of miR-320a or integrin β3 in SACC patients**

**A. Correlation among clinicopathologic status and the expression of miR-320a or integrin β3 in SACC patients of Sun Yat-sen University**						
**Characteristics**	**miR-320a (%)**			**Integrin β3 (%)**		
	**No. of low expression**	**No. of high expression**	***P***	**No. of low expression**	**No. of high expression**	***P***
Sex			0.905			0.097
Male	83 (61.9)	51 (38.1)		45 (33.6)	89 (66.4)	
Female	106 (63.1)	62 (36.9)		73 (43.5)	95 (56.5)	
Age			0.153			0.814
<50	82 (58.2)	59 (41.8)		54 (38.3)	87 (61.7)	
≥50	107 (66.5)	54 (33.5)		64 (39.8)	97 (60.2)	
Tumor Size			0.801			0.319
T1-2	125 (61.9)	77 (38.1)		83 (41.1)	119 (58.9)	
T3-4	64 (64.0)	36 (36.0)		35 (35.0)	65 (65.0)	
Node metastasis			0.029			0.032
N0	154 (59.9)	103 (40.1)		107 (41.6)	150 (58.4)	
N1	35 (77.8)	10 (22.2)		11 (24.4)	34 (75.6)	
TNM stage			0.074			0.044
I-II	95 (57.9)	69 (42.1)		73 (44.5)	91 (55.5)	
III-IV	94 (68.1)	44 (31.9)		45 (32.6)	93 (67.4)	
Distant Metastasis			<0.001			0.001
No	90 (52.3)	82 (47.7)		81 (47.1)	91 (52.9)	
Yes	99 (76.2)	31 (23.8)		37 (28.5)	93 (71.5)	
Status			0.002			0.009
Survival	84 (54.2)	71 (45.8)		72(46.5)	83 (53.5)	
Death	105 (71.4)	42 (28.6)		46(31.3)	101 (68.7)	
**B. Correlation among clinicopathologic status and the expression of miR-320a or integrin β3 in SACC patients of Central South University**						
Sex			0.496			0.865
Male	53 (66.2)	27 (33.8)		29 (36.2)	51 (63.8)	
Female	41 (60.3)	27 (39.7)		26 (38.2)	42 (61.8)	
Age			0.170			0.609
<50	47 (58.0)	34 (42.0)		32 (39.5)	49 (60.5)	
≥50	47 (70.1)	20 (29.9)		23 (34.3)	44 (65.7)	
Tumor Size			0.601			0.861
T1-2	60 (65.2)	32 (34.8)		35 (38.0)	57 (62.0)	
T3-4	34 (60.7)	22 (39.3)		20 (35.7)	36 (64.3)	
Node metastasis			0.811			0.469
N0	80 (63.0)	47 (37.0)		49 (38.6)	78 (61.4)	
N1-N2	14 (66.7)	7 (33.3)		6 (28.6)	15 (71.4)	
TNM stage			0.392			0.608
I-II	56 (66.7)	28 (33.3)		33 (39.3)	51 (60.7)	
III-IV	38 (59.4)	26 (40.6)		22 (34.4)	42 (65.6)	
Distant Metastasis			0.001			0.004
No	42 (51.2)	40 (48.8)		39 (47.6)	43 (52.4)	
Yes	52 (78.8)	14 (21.2)		16 (24.2)	50 (75.8)	
Status			0.004			0.017
Survival	37 (51.4)	35 (48.6)		34 (47.2)	38 (52.8)	
Death	57 (75.0)	19 (25.0)		21 (27.6)	55 (72.4)	

Furthermore, we generated Kaplan-Meier curves to evaluate the correlation between miR-320a expression and metastasis and the survival of the patients. The cumulative metastasis rate up to 10 years was significantly lower for patients with high miR-320a expression from the affiliated hospitals of Sun Yat-sen University than for those with low miR-320a expression (Figure 5D, 27.4% vs. 52.4%, *P* < 0.001). The cumulative survival rate was 62.8% for patients with high miR-320a expression, whereas it was only 44.4% for those with low miR-320a expression (Figure 5E, *P* = 0.001). Similar results were obtained for patients from the affiliated hospitals of Central South University. The cumulative metastasis rate up to 10 years was 25.9% for patients with high miR-320a expression, as opposed to 55.9% for those with low miR-320a expression (Figure 5F, *P* = 0.001), and the cumulative survival rate was 64.8% for patients with high miR-320a expression versus only 39.4% for those with low miR-320a expression (Figure 5G, *P* = 0.003). In contrast, high integrin β3 expression was associated with a high metastasis rate (Additional file 6A and C) and poor survival in the patients (Additional file 6B and D).

To determine whether miR-320a is an independent prognostic covariate for SACCs, we carried out a multivariate Cox proportional hazards analysis (Additional file 7). In the final multivariate Cox regression model, miR-320a expression in SACCs is associated with low lung metastasis (hazard ratio [HR] = 0.45, *P* < 0.001 for patients from the affiliated hospitals of Sun Yat-sen University; HR = 0.363, *P* = 0.001 for patients from the affiliated hospitals of Central South University), independent of other clinical covariates, suggesting that miR-320a can be used as an independent indicator for lung metastasis in SACC patients.

## Discussion

Dysregulation of miRNAs has been well documented in nearly all types of human malignancies, and numerous miRNAs are involved in tumor formation and progression by regulating the expression and action of many oncogenes and tumor suppressor genes. Previously, miR-320a was shown to be downregulated in colon cancer tissues and cancer cell lines, and ectopic expression of miR-320a suppressed the growth of colon cancer cells by directly targeting β-catenin [24]. In leukemia cells, enforced miR-320a expression suppresses transferrin receptor 1 expression and cell proliferation [25]. Furthermore, the miR-320a expression levels were significantly decreased in liver metastasis tissues compared with matched primary colorectal cancer tissues [26]. In our study, CCK8 assays and Annexin V/PI assays demonstrated that miR-320a expression did not influence the proliferation and apoptosis of SACC cells (data not shown). *In vivo* experiments also illustrated that miR-320a did not regulate tumor growth in ACC xenografts. However, reduced miR-320a expression is critical for the invasiveness of SACC cells, and ectopic miR-320a expression represses SACC tumor metastasis by silencing *ITGB3*. These results indicate that miR-320a primarily regulates the metastasis of SACCs. In addition, our study suggested that miR-320a exerts its anti-metastatic function by targeting *ITGB3*, a previously unidentified miR-320a target. This might explain the various functions of miR-320a among different cancer types while also suggesting that distinct features of SACC cells require careful consideration when developing a therapeutic strategy.

Local invasion and distant metastasis are the main causes of death in SACC patients. Therefore, determining the mechanisms that govern the metastasis of SACCs is essential for the development of novel therapeutic strategies to improve patient survival. In the present study, we found that ectopic miR-320a expression represses the invasiveness of SACC cells and the metastasis of SACC xenograft tumors, suggesting that miR-320a may be an effective therapeutic target. In contrast to artificially synthetic siRNAs, microRNAs are endogenous molecules that exist in normal cells, which may minimize their unexpected off-target silencing effects [27,28]. In addition, because a microRNA molecule targets a set of coding genes rather than a single gene, therapies based on microRNA interference could enable more potent cancer treatments by targeting multiple molecular pathways.

In routine clinical practice, the TNM staging system is the key prognostic determinant for patients with salivary adenoid cystic carcinoma. However, large variations in the clinical outcomes of patients with the same cancer stage have been reported, suggesting that the present staging system is not adequate for prognosis. Here, we developed a miRNA signature that was predictive of metastasis in SACC patients, independent of TNM stage. TNM staging is performed mainly on the basis of anatomical information; conversely, the miRNA signature could show the biological characteristics of the SACCs. Identifying miRNAs in patients using quantitative RT-PCR might be a straightforward and clinically applicable procedure. Furthermore, microRNAs are relatively stable compared with other biological macromolecules. miRNAs can be well preserved in tissue samples, even after formalin-fixation and paraffin-embedding, and they can be efficiently extracted and evaluated [29]. Moreover, microRNAs released from tumor cells are protected in membrane-derived exosomes and are thus stable in various bodily fluids, including serum and plasma. Alterations to the microRNA profile in many types of bodily fluids may reflect potential physiological and/or pathological conditions [30,31]. All specimens in our study were obtained from patients in China, and additional sets of independent samples from non-Asian patients will be needed to confirm our findings.

## Conclusions

In summary, we found that miR-320a was downregulated in metastatic SACC cells, enabling the overexpression of its target *ITGB3*. Upregulated *ITGB3* contributed to enhanced cell attachment, invasion and cancer metastasis. These results suggest that miR-320a is an effective metastasis inhibitor for SACC. We also identified a novel miR-320a target, *ITGB3*, in SACC cells, which may be a distinct feature of SACCs. Finally, miR-320a serves as a metastasis prediction marker independent of TNM staging. These findings provide a strong rationale for the potential use of miR-320a as a therapeutic target and prognostic biomarker. The findings of this study were summarized as an illustration (Additional file 8).

## Methods

### Cell culture

The human salivary adenoid cystic carcinoma cell lines ACC2 and ACCM were purchased from the Type Culture Collection of the Chinese Academy of Sciences (Shanghai, China). SACC-83 and SACC-LM were purchased from Peking University (Beijing, China). ACCM and SACC-LM are highly metastatic cells derived from lung metastases of ACC2 and SACC-83 xenografts, respectively [32,33]. All cells were cultivated in RPMI-1640 medium (Gibco, Rockville, MD) supplemented with 10% FBS (Invitrogen, Carlsbad, CA).

### MiRNA microarray analysis

Microarray analyses were performed in ACC2 and ACCM cells as described previously [34]. A heat map demonstrating the average levels of microRNAs, which are differentially expressed in ACCM vs. ACC2, was created using DMVS 2.0 software (Chipscreen Biosciences, Shenzhen, China). The differentially expressed microRNAs are listed in Supplementary Table 1.

### Transfection

All miRNA mimics and antisense oligonucleotides (ASOs) for miRNA were obtained from GenePharma (Shanghai, China). Cells were transfected with 30 nM miRNA mimics or ASOs using Lipofectamine 2000 (Invitrogen). The EmGFP-miR-320a plasmid was also obtained from GenePharma, and blasticidin (Sigma, St Louis, MO) was used to select transfected ACCM cells. *ITGB3* cDNA carrying a wild-type 3′-UTR or a 3′-UTR containing mutated seed sequence for miR-320a (*ITGB3* mut) were cloned into pcDNA 3.1 for “rescue” experiments.

### Quantitative RT-PCR

Real-time PCR was performed using a LightCycler 480 (Roche, Basel, Switzerland). Reactions were run in triplicate in three independent experiments. qRT-PCR for miRNA was performed using the Real-time PCR Universal Reagent (GenePharma). U6 was used as an internal control.

### Western blot analysis

Protein extracts were resolved via 8% SDS-PAGE, transferred onto polyvinylidene difluoride membranes (BioRad, Berkeley, CA), probed with antibodies against human integrin β3 (Abcam, Cambridge, UK), p-FAK (Y397), FAK, p-Src (Y416), Src (Cell signaling, Boston, MA) or β-actin (Proteintech, Chicago, IL) followed by a peroxidase-conjugated secondary antibody (Proteintech), and then visualized by chemiluminescence (ImageQuant RT ECL, GE, Fairfield, CT).

### Adhesion assay

Fibronectin-coated 24-well plates (Corning, New York, NY) were seeded with, 1 × 10^4^ cells/well and incubated for 15 min, after which the non-adherent cells were washed away and the adherent cells were fixed in 4% paraformaldehyde, stained with crystal violet and counted (5 random 100× fields per well). Three independent experiments were performed, and the data are presented as the average ± SD [35].

### Transwell assay

A total of 1 × 10^5^ cells were seeded into the upper chamber of a polycarbonate transwell filter chamber (Corning) and incubated for 22 h. For the invasion assays, the upper chamber was coated with Basement Membrane (R&D, Minneapolis, MN). Cells on the lower membrane surface were fixed in 4% paraformaldehyde, stained with crystal violet and counted (5 random 100× fields per well). Three independent experiments were performed and the data are presented as the average ± SD.

### Luciferase reporter assay

We cloned the miR-320a response element (wild type or mutant) of the 3′-UTR of *ITGB3* into the pMIR-REPORT plasmid downstream of the luciferase reporter gene. Luciferase activity was assayed using a luciferase assay kit (Promega, Madison, WI), and the target effect was expressed as the relative luciferase activity of the reporter vector with the target sequence over that of the vector without the target sequence.

### Immunofluorescence staining

Cells were stained for immunofluorescence on coverslips. After fixation and permeabilization, the cells were incubated with primary antibodies against integrin β3 or FITC-phalloidin (Sigma) and then incubated with Alex 555-conjugated secondary antibodies (Invitrogen). The coverslips were counterstained with DAPI and imaged under a TCS SP5 confocal microscope (Leica, Solms, Germany).

### Tumor xenografts

A total of 5 × 10^6^ ACC2 or ACCM cells, either untransduced or transduced with the miRNA-expressing vector EmGFP-miR-320a, were injected into the salivary site of 5-week-old BALB/c-nu mice. After tumors were detected, the tumor size was measured and calculated: Volume (mm^3^) = length × width^2^ × 0.5. Tumor xenografts, as well as whole lung and liver tissues, were then harvested, weighed and snap-frozen in liquid nitrogen. To evaluate *in vivo* metastasis, fluorescence images of whole mice or their lungs and livers were acquired using an IVIS Lumina Imaging System (Xenogen, Alameda, CA), and portions of the lung and liver tissues were used for qRT-PCR for hHPRT (human hypoxanthine-guanine phosphoribosyltransferase) expression. Cryosections (4 μm) were stained with hematoxylin and eosin (HE) and used for immunohistochemistry. The procedures were approved by Sun Yat-sen University Animal Care and Use Committee.

### Patients and tissue samples

Two independent retrospective SACC patient cohorts were studied, including 302 patients from the affiliated hospitals of Sun Yat-sen University, such as Sun Yat-sen Memorial Hospital, the First Affiliated Hospital and the Hospital of Stomatology (Guangzhou, China), and 148 patients from the affiliated hospitals of Central South University, such as Xiangya Stomatological Hospital, the Second Xiangya Hospital (Changsha, China) between Jan 1, 1999, and Dec 31, 2008. None of the patients received any chemotherapy or radiotherapy prior to surgery. The tumors were staged according to the TNM staging system. The tissues were obtained from the respective pathology departments, and histological diagnosis and scoring of all the cases were performed by two independent pathologists (Yanyang Chen and Yang Li). Survival time was calculated from the date of surgery to the date of death or to the last follow-up. The date of death was obtained from patient records or through follow-up telephone calls. This study was approved by the institutional ethical review boards of both hospitals, and written informed consent was obtained from all patients.

### In situ hybridization

This assay was performed according to the manufacturer’s protocol (Exiqon, Vedbaek, Denmark). Briefly, after demasking, microRNA was hybridized to 5′-DIG-labeled LNA™ probes. Then, the digoxigenin was recognized by a specific anti-DIG antibody that is directly conjugated to alkaline phosphatase. The nuclei were counterstained with hematoxylin.

### Immunohistochemistry

For immunohistochemistry [36], the samples were incubated with an integrin β3 antibody at 4°C overnight. The sections were then treated with a secondary antibody, followed by further incubation with a streptavidin-horseradish peroxidase complex. Diaminobenzidine (Dako, Carpinteria, CA) was used as a chromogen, and the nuclei were counterstained with hematoxylin.

### Scoring of ISH and IHC

In each section, 5 fields of 200 tumor cells were counted randomly, and the scores for miR-320a and integrin β3 were determined by combining the proportion of positively stained tumor cells and the intensity of staining. The proportion of positively stained tumor cells was graded from 0 to 3 (0, <5% positive cells; 1, 5-25%; 2, 26-50%; 3, >50%). The intensity of staining was recorded on a scale of 0 (no staining), 1 (weak staining, light blue or yellow), 2 (moderate staining, blue or yellow), and 3 (strong staining, dark blue or yellow). For tumors that showed heterogeneous staining, the predominant pattern was taken into account for scoring. The staining index (SI) was calculated as follows: staining index = proportion of positively stained tumor cells × staining intensity. Using this method, the expression of miR-320a and integrin β3 was evaluated by the SI and scored as 0, 1, 2, 3, 4, 6, or 9. A composite score greater than the median value was considered to be high expression, and composite scores less than or equal to the median value were considered to be low expression.

### Statistical analysis

All statistical analyses were performed using SPSS 17.0 software for Windows (SPSS Inc., Chicago, IL). The Chi-squared test was used to analyze the relationship between miR-320a or integrin β3 expression and clinicopathologic characteristics. To measure the association between pairs of variables, Spearman order correlations were run. Kaplan-Meier survival curves were plotted, and the log-rank test was performed. The significance of various variables for lung metastasis was analyzed by the Cox proportional hazards model in a multivariate analysis. All experiments with cell cultures were performed at least in triplicate. The results are expressed as the mean ± SD. *P* < 0.05 was considered statistically significant.

## Additional files

Additional file 1:
**MicroRNA microarray analysis comparing ACC2 and ACCM cells.**
Additional file 2:
**The differential expression of miRNAs in the ACCM and ACC2 cells was validated using qRT-PCR analysis.** U6 was used as an internal control. Additional file 3:
**Reduction of miR-320a promotes the invasiveness of SACC cells.** (A) miR-320a expression in SACC cells was determined using qRT-PCR analysis. **P* < 0.05; ***P* < 0.01 vs. mock transfection. U6 was used as an internal control. An adhesion assay (B) and transwell assay (C, D) showed that the adhesion, invasion and migration of ACC2 and SACC-83 cells were enhanced by miR-320a ASO (100x). (E) Quantification of the adhesive, invasive and migratory cells assessed using the adhesion and transwell assays. **P* < 0.05; ***P* < 0.01 vs. mock. Additional file 4:
**MiR-320a suppresses integrin β3 expression in ACCM cells implanted in BALB/c-nu mice.** (A) Transfection with the EmGFP-320a vector specifically enhances miR-320a expression in ACCM cells as determined by qRT-PCR. (B) Tumor volumes of mice inoculated with ACC2 cells or ACCM cells stably expressing miR-320a. (C) HE staining (200x) of the tumor and immunohistochemical staining (400x) for integrin β3 and PCNA. The white arrows indicate focal metastases. The percentages of integrin β3 (D) and PCNA (E) positive cells in the tumor section are analyzed. ***P* < 0.01 vs. ACC2 cells. ^##^ *P* < 0.01 vs. EmGFP transfection. Additional file 5:
**MiR-320a expression was negatively correlated with integrin β3 expression in SACCs.** Associations between miR-320a expression and integrin β3 expression in SACC samples from affiliated hospitals of Sun Yat-sen University (A) or from affiliated hospitals of Central South University (B) were analyzed using Spearman’s rank order correlation coefficient. Additional file 6:
**High expression of integrin β3 indicates poor patient survival and high risk of SACC metastasis.** Kaplan-Meier curves for SACC patients from affiliated hospitals of Sun Yat-sen University plotted according to integrin β3 expression and metastasis difference (A) or plotted according to integrin β3 expression and survival difference (B) were analyzed using the log rank test. Kaplan-Meier curves for SACC patients from affiliated hospitals of Central South University plotted according to integrin β3 expression and metastasis difference (C) or plotted according to integrin β3 expression and survival difference (D). The median level was used to distinguish high expression and low expression. Additional file 7:
**Multivariate analysis of various variables for lung metastasis in SACC patients using Cox regression analysis.**
Additional file 8:
**Schematic summary of the findings of this study.**
