# Supplementary material for: MiR-320a acts as a prognostic factor and Inhibits metastasis of salivary adenoid cystic carcinoma by targeting ITGB3
Source: Mol Cancer. 2015 Apr 29;14:96. doi: 10.1186/s12943-015-0344-y (PMC4423101; doi:10.1186/s12943-015-0344-y)
Supplement: Additional file 1: — MicroRNA microarray analysis comparing ACC2 and ACCM cells. [file 12943_2015_344_MOESM1_ESM.pdf]

Additional file 1. MicroRNA microarray analysis comparing ACC2 and ACCM cells

microRNA Name	Ratio (ACCM: ACC2)
hsa-let-7g	2.41
hsa-miR-24	2.03
hsa-miR-195	4.83
hsa-miR-342-3p	2.07
hsa-miR-939	2.63
hsa-let-7b	0.49
hsa-miR-23b	0.48
hsa-miR-93	0.44
hsa-miR-98	0.45
hsa-miR-320a	0.50
hsa-miR-674	0.40
hsa-miR-886-5p	0.48
