# Supplementary figures and images for: MiR-320a acts as a prognostic factor and Inhibits metastasis of salivary adenoid cystic carcinoma by targeting ITGB3
Source: Mol Cancer. 2015 Apr 29;14:96. doi: 10.1186/s12943-015-0344-y (PMC4423101; doi:10.1186/s12943-015-0344-y)

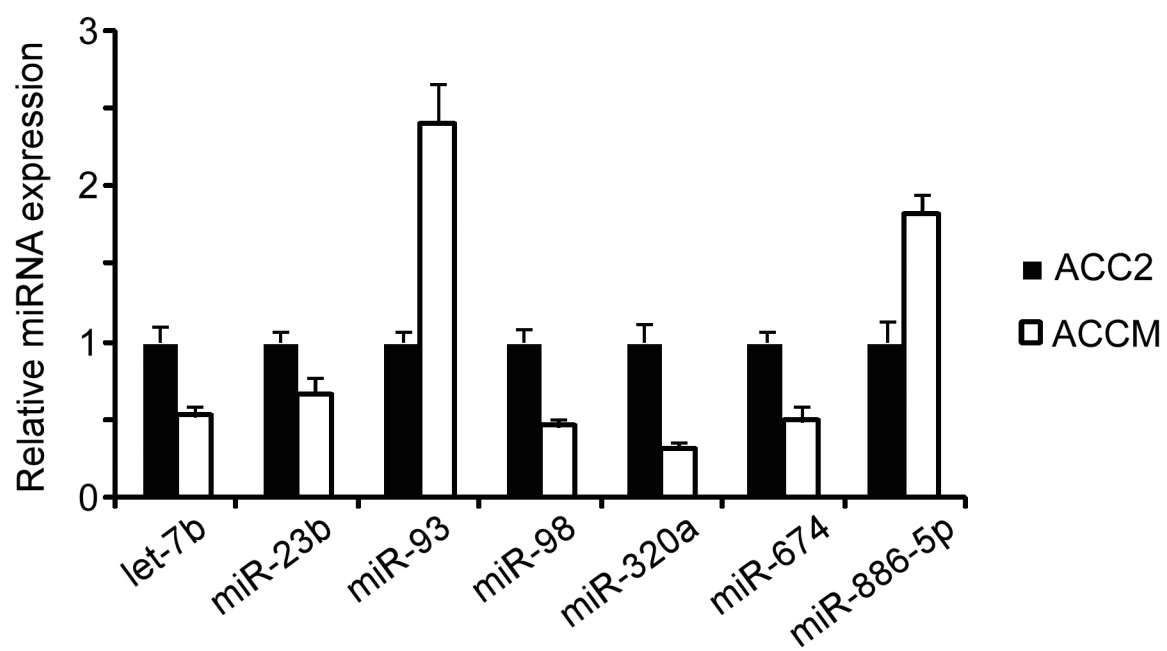

Supplement: Additional file 2: — The differential expression of miRNAs in the ACCM and ACC2 cells was validated using qRT-PCR analysis. U6 was used as an internal control. [file 12943_2015_344_MOESM2_ESM.pdf]

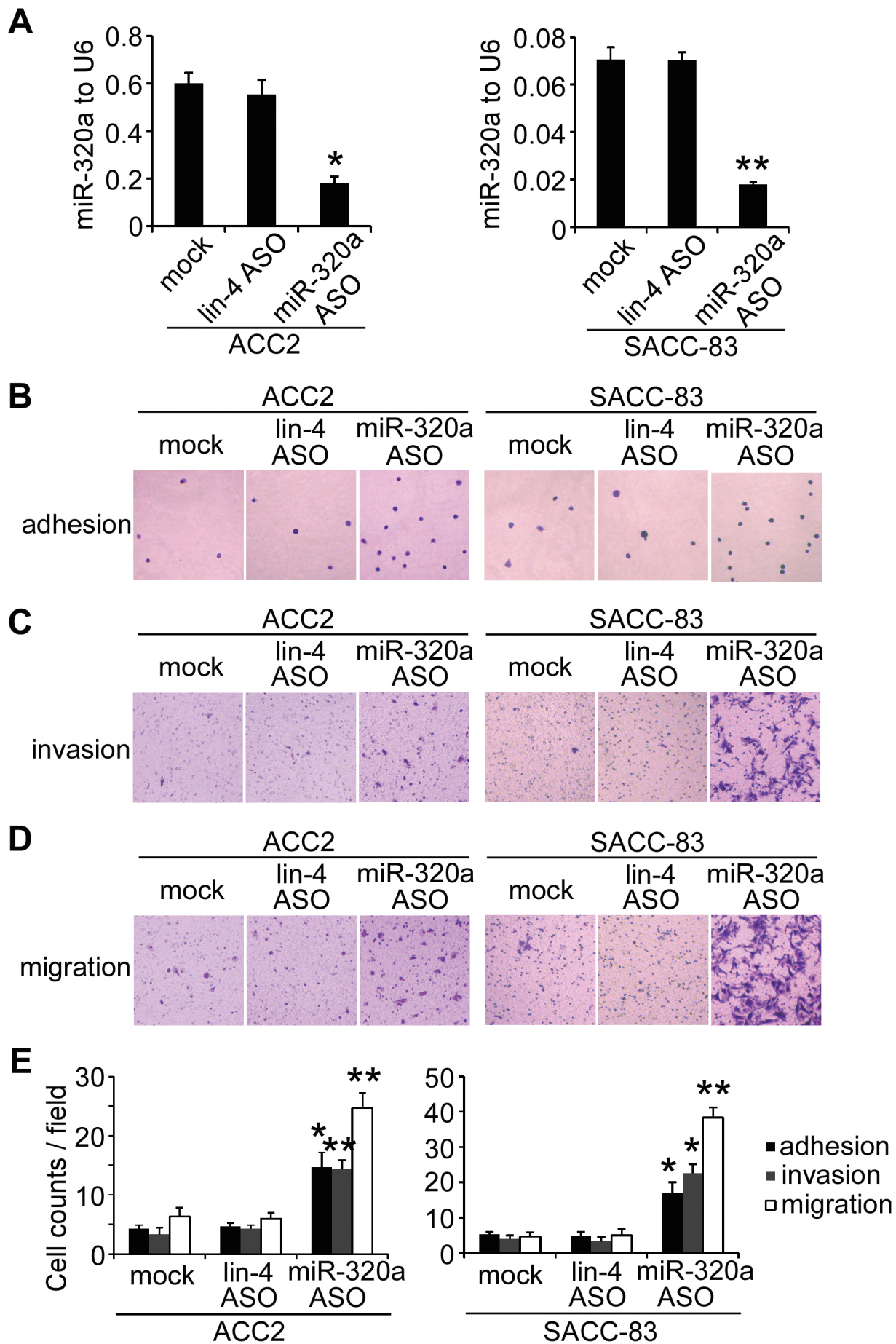

Supplement: Additional file 3: — Reduction of miR-320a promotes the invasiveness of SACC cells. (A) miR-320a expression in SACC cells was determined using qRT-PCR analysis. *P < 0.05; **P < 0.01 vs. mock transfection. U6 was used as an internal control. An adhesion assay (B) and transwell assay (C, D) showed that the adhesion, invasion and migration of ACC2 and SACC-83 cells were enhanced by miR-320a ASO (100x). (E) Quantification of the adhesive, invasive and migratory cells assessed using the adhesion and transwell assays. *P < 0.05; **P < 0.01 vs. mock. [file 12943_2015_344_MOESM3_ESM.pdf]

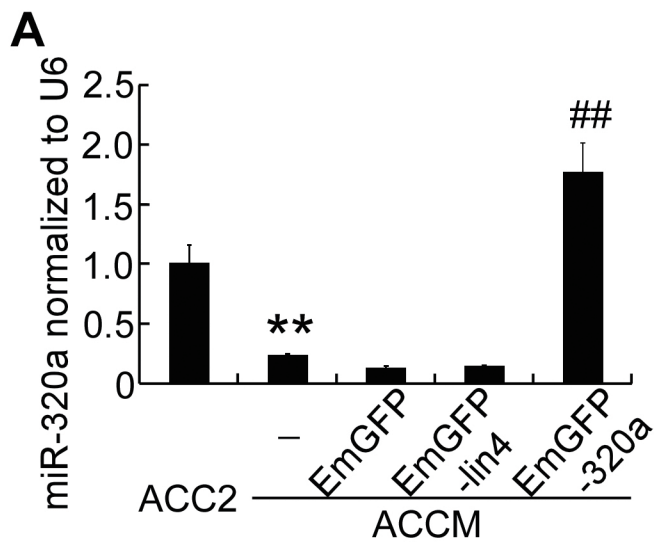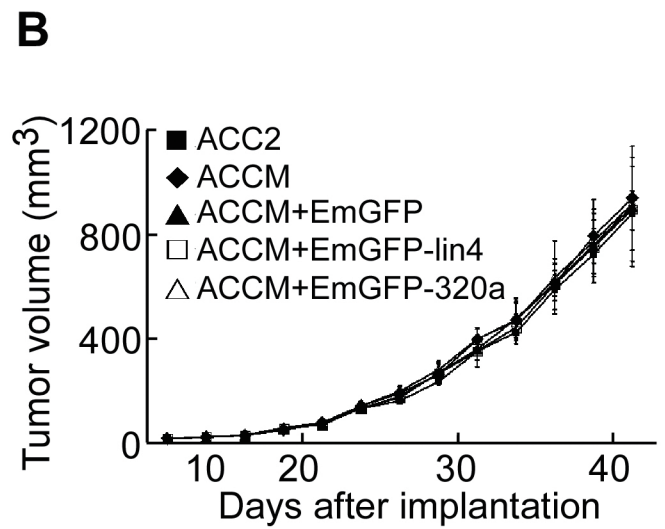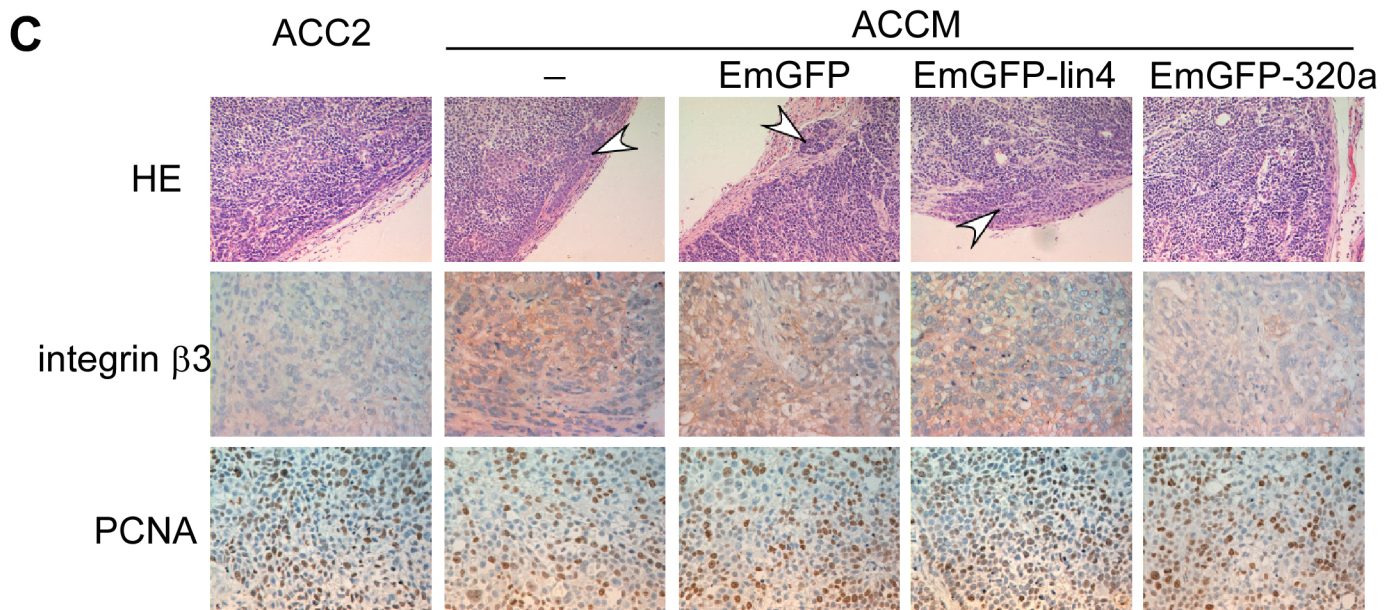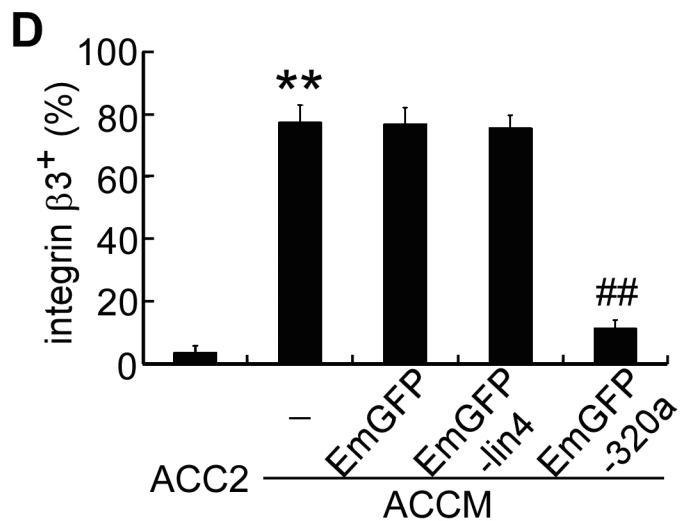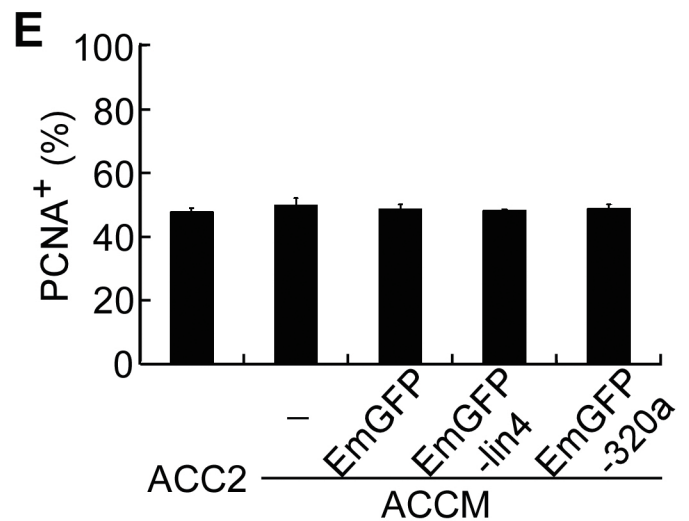

Supplement: Additional file 4: — MiR-320a suppresses integrin β3 expression in ACCM cells implanted in BALB/c-nu mice. (A) Transfection with the EmGFP-320a vector specifically enhances miR-320a expression in ACCM cells as determined by qRT-PCR. (B) Tumor volumes of mice inoculated with ACC2 cells or ACCM cells stably expressing miR-320a. (C) HE staining (200x) of the tumor and immunohistochemical staining (400x) for integrin β3 and PCNA. The white arrows indicate focal metastases. The percentages of integrin β3 (D) and PCNA (E) positive cells in the tumor section are analyzed. **P < 0.01 vs. ACC2 cells. ## P < 0.01 vs. EmGFP transfection. [file 12943_2015_344_MOESM4_ESM.pdf]

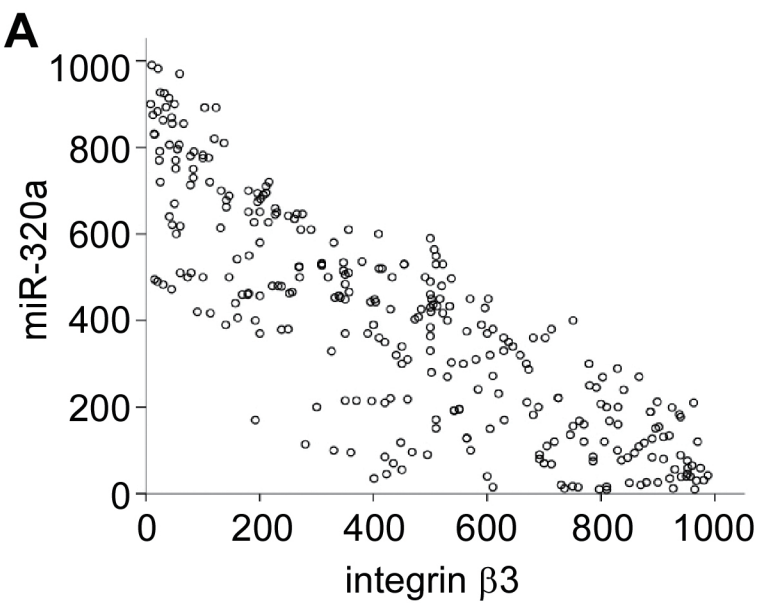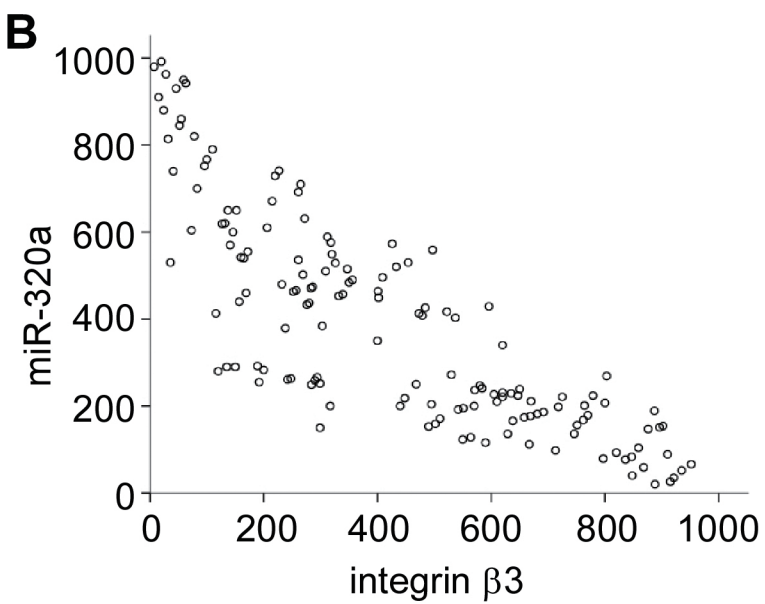

Supplement: Additional file 5: — MiR-320a expression was negatively correlated with integrin β3 expression in SACCs. Associations between miR-320a expression and integrin β3 expression in SACC samples from affiliated hospitals of Sun Yat-sen University (A) or from affiliated hospitals of Central South University (B) were analyzed using Spearman’s rank order correlation coefficient. [file 12943_2015_344_MOESM5_ESM.pdf]

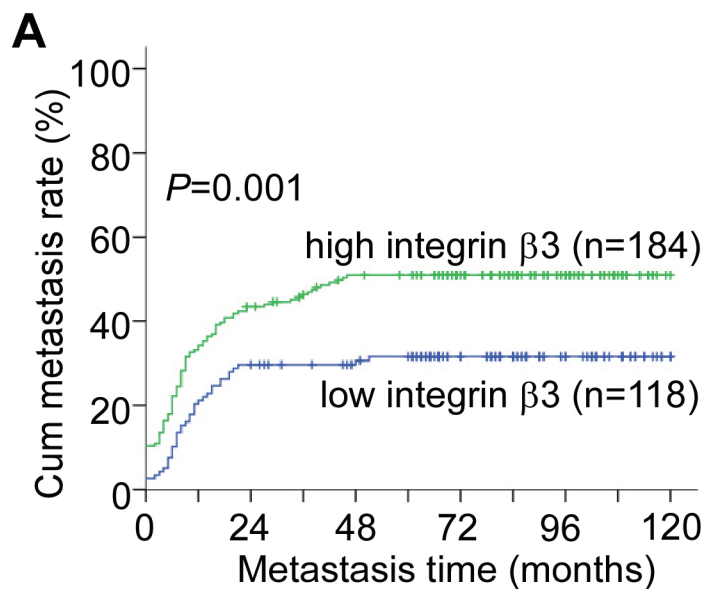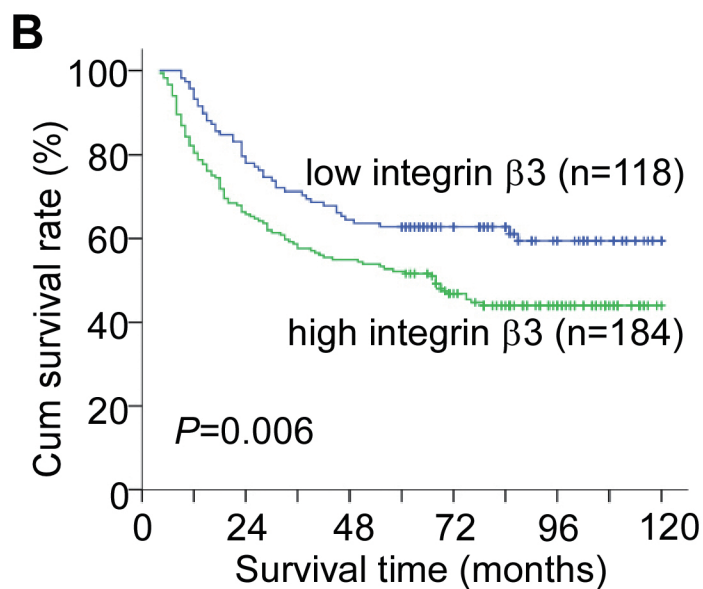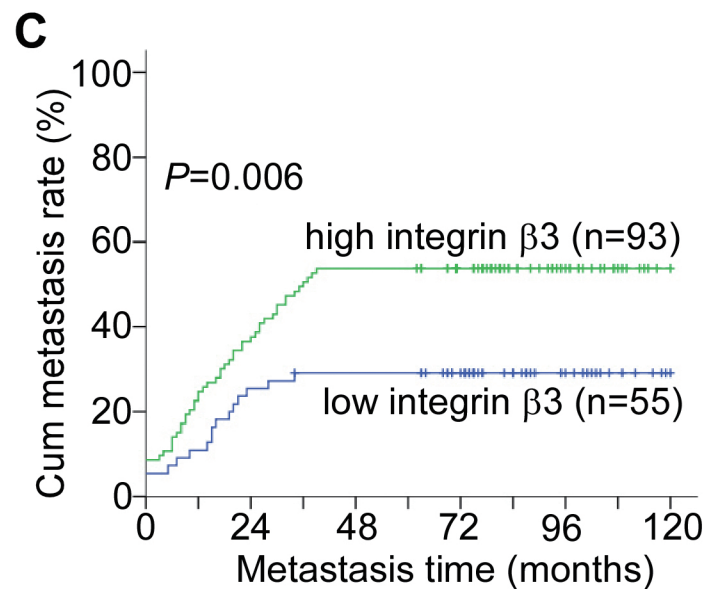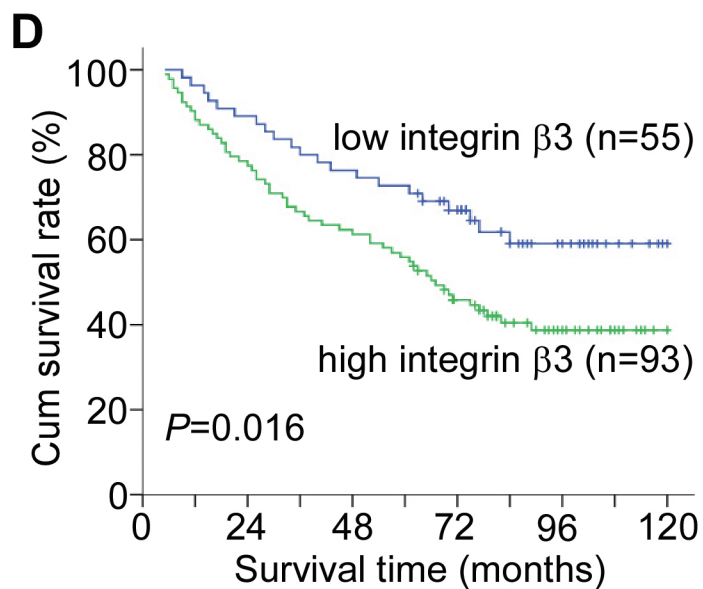

Supplement: Additional file 6: — High expression of integrin β3 indicates poor patient survival and high risk of SACC metastasis. Kaplan-Meier curves for SACC patients from affiliated hospitals of Sun Yat-sen University plotted according to integrin β3 expression and metastasis difference (A) or plotted according to integrin β3 expression and survival difference (B) were analyzed using the log rank test. Kaplan-Meier curves for SACC patients from affiliated hospitals of Central South University plotted according to integrin β3 expression and metastasis difference (C) or plotted according to integrin β3 expression and survival difference (D). The median level was used to distinguish high expression and low expression. [file 12943_2015_344_MOESM6_ESM.pdf]

normal salivary epithelia

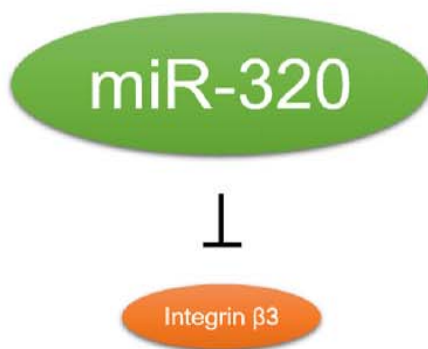

Salivary Adenoid Cystic Carcinoma (SACC)

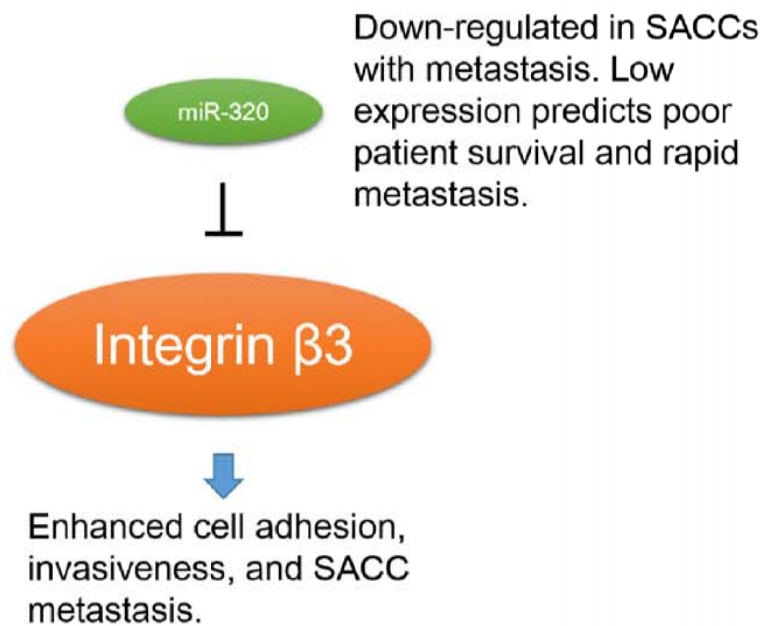

Supplement: Additional file 8: — Schematic summary of the findings of this study. [file 12943_2015_344_MOESM8_ESM.pdf]
