# Supplementary material for: MiR-320a acts as a prognostic factor and Inhibits metastasis of salivary adenoid cystic carcinoma by targeting ITGB3
Source: Mol Cancer. 2015 Apr 29;14:96. doi: 10.1186/s12943-015-0344-y (PMC4423101; doi:10.1186/s12943-015-0344-y)
Supplement: Additional file 7: — Multivariate analysis of various variables for lung metastasis in SACC patients using Cox regression analysis. [file 12943_2015_344_MOESM7_ESM.pdf]

Additional file 7. Multivariate analysis of various variables for lung metastasis in SACC patients using Cox regression analysis

Variables	Sun Yat-sen University (N=302)			Central South University (N=148)		
	HR	(95% CI)	*P*	HR	(95% CI)	*P*
Gender (female vs. male)	1.249	(0.877-1.778)	0.217	0.925	（0.568-1.508）	0.756
Age (≥50 vs.＜50)	0.780	(0.551-1.104)	0.161	1.004	（0.604-1.668）	0.989
Tumor diameter (≥4cm vs.＜4cm)	1.177	（0.500-2.768）	0.709	1.151	（0.376-3.524）	0.805
Lymph node status (N_1 vs. N_0)	1.595	（0.987-2.576）	0.056	2.077	（1.056-4.086）	0.034
TNM stage (III-IV vs. I-II)	1.400	（0.943-2.079）	0.095	1.630	（0.925-2.870）	0.091
Integrin β3 expression (high vs. low)	1.325	（0.861-2.039）	0.200	1.334	（0.712-2.499）	0.369
MiR-320a expression (high vs. low)	0.450	（0.300-0.675）	< 0.001	0.363	（0.200-0.659）	0.001
